# Test–retest reproducibility of cannabinoid-receptor type 1 availability quantified with the PET ligand [^11^C]MePPEP

**DOI:** 10.1016/j.neuroimage.2014.04.020

**Published:** 2014-08-15

**Authors:** Daniela A. Riaño Barros, Colm J. McGinnity, Lula Rosso, Rolf A. Heckemann, Oliver D. Howes, David J. Brooks, John S. Duncan, Federico E. Turkheimer, Matthias J. Koepp, Alexander Hammers

**Affiliations:** a Centre for Neuroscience, Department of Medicine, Imperial College London, London, UK; b MRC Clinical Sciences Centre Hammersmith Hospital, London, UK; c Department of Clinical and Experimental Epilepsy, Institute of Neurology, University College London, UK; d Epilepsy Society, Chalfont St Peter, UK; e Neurodis Foundation, CERMEP, Imagerie du Vivant, Lyon. France; f Institute of Clinical Medicine, Aarhus University, Denmark; g Centre for Neuroimaging, Institute of Psychiatry, King's College London, London, UK

**Keywords:** CB_1_, Positron Emission Tomography, Reliability, Intra-class correlation coefficient

## Abstract

**Background:**

Endocannabinoids are involved in normal cognition, and dysfunction in cannabinoid-receptor-mediated neurotransmission has been suggested in a variety of neurological and psychiatric pathologies. The type 1 cannabinoid receptor (CB_1_) is widely expressed in the human central nervous system. The objective of this study was to quantify the test–retest reproducibility of measures of the PET ligand [^11^C]MePPEP in order to assess the stability of CB_1_-receptor quantification in humans in vivo.

**Methods:**

Fifteen healthy subjects (eight females; median age 32 years, range 25 to 65 years) had a 90-minute PET scan on two occasions after injection of a median dose of [^11^C]MePPEP of 364 MBq. Metabolite-corrected arterial plasma input functions were obtained for all scans. Eight ROIs, reflecting different levels of receptor densities/concentrations, were defined automatically: hippocampus, anterior cingulate gyrus, inferior frontal gyrus, caudate nucleus, globus pallidus, nucleus accumbens, thalamus, and pons. We used seven quantification methods: reversible compartmental models with one and two tissue classes, two and four rate constants, and a variable blood volume term (2kbv; 4kbv); model-free (spectral) analyses with and without regularisation, including one with voxel-wise quantification; the simplified reference tissue model (SRTM) with pons as a pseudo-reference region; and modified standard uptake values (mSUVs) calculated for the period of ~ 30–60 min after injection. Percentage test–retest change and between-subject variability were both assessed, and test–retest reliability was quantified by the intraclass correlation coefficient (ICC). The ratio of binding estimates pallidum:pons served as an indicator of a method's ability to reflect binding heterogeneity.

**Results:**

Neither the SRTM nor the 4kbv model produced reliable measures, with ICCs around zero. Very good (> 0.75) or excellent (> 0.80) ICCs were obtained with the other methods. The most reliable were spectral analysis parametric maps (average across regions ± standard deviation 0.83 ± 0.03), rank shaping regularised spectral analysis (0.82 ± 0.05), and the 2kbv model (0.82 ± 0.09), but mSUVs were also reliable for most regions (0.79 ± 0.13). Mean test–retest changes among the five well-performing methods ranged from 12 ± 10% for mSUVs to 16% for 2kbv. Intersubject variability was high, with mean between-subject coefficients of variation ranging from 32 ± 13% for mSUVs to 45% for 2kbv. The highest pallidum:pons ratios of binding estimates were achieved by mSUV (4.2), spectral analysis-derived parametric maps (3.6), and 2kbv (3.6).

**Conclusion:**

Quantification of CB_1_ receptor availability using [^11^C]MePPEP shows good to excellent reproducibility with several kinetic models and model-free analyses, whether applied on a region-of-interest or voxelwise basis. Simple mSUV measures were also reliable for most regions, but do not allow fully quantitative interpretation. [^11^C]MePPEP PET is well placed as a tool to investigate CB_1_-receptor mediated neurotransmission in health and disease.

## Highlights

• [11C]MePPEP is a PET tracer for cannabinoid receptors (CB1R).
• Extensive evaluation of [11C]MePPEP data quantification strategies in large sample
• We highlight successful methods to quantify CB1R in regions of interest.
• Highly reliable parametric maps (ICC 0.83 ± 0.03) allow whole-brain surveys.
• Modified standard uptake values also reliable, without arterial input functions

## Graphical abstract

Cannabinoid receptor concentrations assessed with [^11^C]MePPEP-PET. Top, reliability (ICCs ± SDs) of different quantification strategies: blue, compartmental models; red, spectral analysis variants; green, SRTM; yellow, modified SUVs. Bottom, parametric *V*_T_ map.

**Figure:**
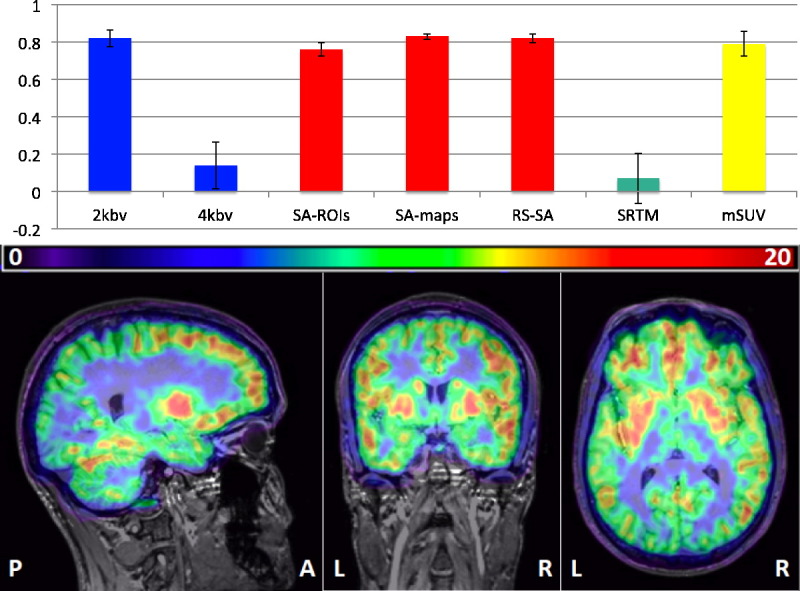

## Introduction

Endocannabinoids and their receptors are involved in a wide spectrum of conditions, e.g. addiction (Bossong et al., 2009) and epilepsy (Goffin et al., 2011), as well as in normal cognition (Hampson et al., 2011).

Two transmembrane G-protein coupled receptor types in the endocannabinoid system have been discovered. Type 1 (CB_1_) is found in the central nervous system (CNS) and in neuronal and non-neuronal tissues outside the CNS (Matsuda et al., 1990). Abundantly expressed in presynaptic glutamatergic and GABAergic terminals (Katona and Freund, 2008), CB_1_ receptors have a heterogeneous CNS distribution. High concentrations are found in the cerebral cortex, hippocampus, caudate nucleus and putamen, substantia nigra pars reticulata, globus pallidus, entopeduncular nucleus, the molecular layer of the cerebellum and in pain pathways of brain and spinal cord (Herkenham et al., 1990, Irving et al., 2002). However, the thalamus and most of the brainstem show low concentrations (Herkenham et al., 1990).

In the preclinical setting, a wide range of CB_1_-selective radioligands have been used successfully in vitro, such as [^3^H]CP-55,940 (Devane et al., 1988, Devane et al., 1992), [^3^H]SR141716A (Petitet et al., 1996), and [^35^S]GTPγS (Griffin et al., 1998); and also in vivo, such as (−)-5′-[^18^F]-Δ^8^-THC (Charalambous et al., 1991), [^123^I]AM251 (Gatley et al., 1996), [^123^I]AM281 (Gatley et al., 1998), [^11^C]OMAR ([^11^C]JHU75528) (Horti et al., 2006), [^11^C]MePPEP (Yasuno et al., 2008), [^11^C]/[^18^F]-PipISB (Finnema et al., 2009) and [^11^C]CB-119 (Hamill et al., 2009). In vivo human brain CB_1_ availability has recently become quantifiable with PET and the use of tracers such as [^18^F]MK-9470 (Burns et al., 2007), [^11^C]OMAR (Wong et al., 2010), [^11^C]MePPEP (Terry et al., 2009), and [^18^F]FMPEP-*d*_2_ (G.E. Terry et al., 2010b).

MePPEP ((*3R*,*5R*)-5-(3-methoxy-phenyl)-3-((*R*)-1-phenyl-ethylamino)-1-(4-trifluoro-methyl-phenyl)-pyrrolidin-2-one) is a CB_1_-selective inverse agonist. [^11^C]MePPEP has high and stable brain uptake in vivo. Despite its moderately high lipophilicity (measured LogD_7.4_ = 4.8) (Yasuno et al., 2008), its specific binding is relatively high with > 85% in monkey brain and 65% determined using CB_1_ knockout mouse brain (Terry et al., 2008, Terry et al., 2010b, Yasuno et al., 2008).

One reproducibility study with [^11^C]MePPEP has been performed in humans so far, involving eight test–retest scans (Terry et al., 2009) after injection of high doses of [^11^C]MePPEP (~ 650 MBq). Only standard uptake values and distribution volumes derived from compartmental modelling were examined.

In the present study we calculated the reproducibility of various parameters to describe CB_1_ receptor availability with [^11^C]MePPEP, including compartmental modelling techniques, spectral analysis variants, a simplified reference tissue model, and simple mSUVs, in regions representative of various CB_1_ receptor concentrations in 15 healthy volunteers.

## Materials and methods

### Subjects

Ethical approval was obtained from the London — Surrey Borders Research Ethics Committee, and permission to undertake the study from the UK's radiation protection agency (ARSAC). Seventeen healthy subjects were recruited and gave written informed consent. Exclusion criteria were: history or presence of psychiatric or systemic medical condition, inability to provide informed consent, claustrophobia, any contraindication for undergoing MR, positive urine drug test, positive urine pregnancy test, general practitioner's (family doctor's) advice against participation, regular medication, use of cannabis within the previous three months or on more than five occasions over the subject's lifetime, and pathological modified Allen's test for patency of the ulnar artery (Allen, 1929, Cable et al., 1999, Slogoff et al., 1983). From this sample two were excluded: one subject with pathological modified Allen's test; another withdrew consent for the retest scan. Hence, a total of fifteen healthy subjects (8 females; median age 32 years, range 25 to 65 years), without history of either systemic medical or psychiatric conditions or substance abuse were scanned twice. Demographic data are detailed in Table 1. All subjects underwent a urine drug screen cassette test for 11-nor-∆^9^-THC, morphine, amphetamine, benzoylecgonine (the main metabolite of cocaine), methamphetamine and oxazepam (Monitect^©^; BMC, California, U.S.A.) prior to PET scanning. All female patients of childbearing age underwent a urine pregnancy test.

**Table 1:** Subjects' demographic and injectate details. BMI = Body Mass Index; Min = minimum; Max = maximum.

Subject no.	Age	Gender	BMI	Scan interval (days)	Dose (MBq)	Radiochemistry purity (%)	Co-injected mass (μg)	Specific activity (MBq/nmol)
1	28	M	23	24	365	98	4.2	39
					364	98	6.2	27
2	44	M	28	23	375	96	3.0	56
					376	99	4.3	40
3	42	M	25	122	356	96	2.3	70
					373	100	2.3	72
4	26	M	24	1	361	98	3.4	48
					366	98	2.7	61
5	31	M	24	19	353	98	4.2	38
					355	99	2.7	60
6	27	F	21	309	367	99	4.6	37
					363	97	1.7	99
7	32	F	36	37	351	100	3.2	50
					316	100	8.3	17
8	65	F	22	23	355	97	2.3	69
					371	96	4.0	42
9	27	M	22	67	369	98	3.9	43
					366	98	3.1	54
10	29	F	23	4	360	97	9.7	17
					364	100	5.8	29
11	57	M	30	30	373	97	2.9	60
					399	96	5.0	37
12	61	F	36	93	361	96	1.8	92
					349	96	1.6	97
13	63	F	29	12	384	97	3.5	50
					380	98	3.3	52
14	25	F	31	156	364	97	2.4	69
					357	100	4.4	37
15	62	F	24	10	362	97	1.4	122
					366	97	3.3	51
Median	32		24	24	364	98	3.3	98
Interquartile range (25th-75th)	27–61		23–30	12–93	356–372	97–99	2.4–4.3	38–69
Min	25		21	1	316	96	1.4	17
Max	65		36	309	399	100	9.7	122

### Radiochemistry

[^11^C]MePPEP was synthesised on site by Hammersmith Imanet following a procedure described previously (Yasuno et al., 2008). Details of the injectate are listed in Table 1.

### PET data acquisition

PET scans were acquired on a Siemens/CTI ECAT EXACT HR + 962 camera (Knoxville, TN, USA) in 3D mode. Ten-minute transmission scans for attenuation correction were obtained prior to dynamic emission scans using a rotating 137Cs point source. Each dynamic acquisition was 90 min long and consisted of 35 frames of increasing length (1 × 30″, 6 × 10″, 3 × 20″, 3 × 30″, 3 × 60″, 6 × 120″, 8 × 300″ and 3 × 600″). 30 s after the scan start, [^11^C]MePPEP was injected as an intravenous bolus injection of ~ 370 MBq (median 364 MBq, range 316–399 MBq; Table 1). Subjects were scanned on two separate days with a median interval of 24 days (range 1 to 309; Table 1).

The head position was maintained throughout and monitored with the camera's positioning laser. If movement was noticed, subjects were repositioned and underwent a second transmission scan at the end of the dynamic scan. To compensate for head movement during dynamic scans, we used a *post hoc* frame-by-frame realignment method, as described later (section “PET data quantification”). Data were reconstructed using FORE (Defrise et al., 1997) and 2D FBP (ramp filter, kernel 2.0 mm FWHM). Voxel sizes of reconstructed images were 2.092 × 2.092 × 2.42 mm.

### Input function derivation

Continuous and intermittent blood samples were collected to allow the subsequent generation of metabolite-corrected arterial plasma time–activity curves (TACs) (Hammers et al., 2008). During the first 15 min blood was withdrawn continuously at a rate of 300 ml/h and radioactivity measured in a BGO detection system (Jones et al., 1994). To quantify plasma and whole blood radioactivity, as well as to allow quantification of the parent fraction of the radiotracer, intermittent discrete (10 ml) samples were taken with heparinised syringes before the scan (baseline) and at the following time points after scan start: 3, 5, 10, 15, 20, 30, and 50 min. At 75 min, a larger 17 ml sample was taken to allow quantification despite radioactivity decay. Parent fraction quantification was not possible at 90 min; hence at this time point only three millilitres was withdrawn for plasma and whole blood radioactivity measurement. Continuous plasma input functions (IF) were derived by cross-calibration and combination of the continuous and discrete data, multiplication with the fitted plasma-over-blood ratio, and correction for parent radiotracer fraction, as described in detail in previous studies (Hammers et al., 2007a, Jones et al., 1994).

### MRI data acquisition, analysis and generation of ROIs

All subjects had 3D T1 weighted MRI scans with approximately millimetric voxel sizes on a Phillips Intera 3 Tesla (3 T) MRI scanner (Best, The Netherlands) at the Robert Steiner MRI Unit, Hammersmith Hospital, for co-registration and ROI definition. There was no visible structural abnormality on any of the T1-weighted images.

T1-weighted images were segmented into tissue classes using the statistical parametrical mapping software SPM8 (Statistical Parametric Mapping, Wellcome Trust Centre for Neuroimaging, UCL, London, www.fil.ion.ucl.ac.uk/spm) under MATLAB© 7.4 (MathWorks).

The T1-weighted images were also anatomically segmented using MAPER (multi-atlas propagation with enhanced registration; Heckemann et al., 2010). Using high-dimensional image registration, 30 MRI data sets, each associated with manually determined labels of 83 regions (Gousias et al., 2008, Hammers et al., 2003), were propagated to the target brain. Label fusion was used to obtain 83 regions of interest (ROIs) in target space (Heckemann et al., 2006).

The T1-weighted images and corresponding MAPER-derived individual segmentations as well as individual grey matter (GM) probability images were co-registered with each subject's corresponding processed PET summation image for test and retest scans separately. For the cortical ROIs, the individual atlases in PET space were then multiplied with the grey matter probability maps thresholded at 0.5 using Analyze© 8.1 biomedical imaging software (Mayo Clinic 2002). These regions of interest were then used to sample the dynamic or parametric images.

### Manual delineation of the pons

**Fig. 1:**
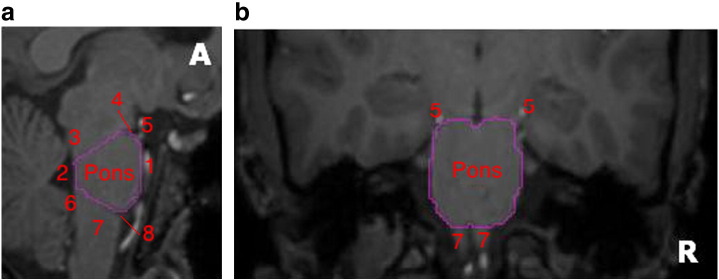
Manual delineation of the pons on MRI. (a) Sagittal view, (b) coronal view, A — Anterior, R — right. The pons was first delineated on sagittal views, followed by coronal and axial views, with the following limits; anterior/ventral: cisterna interpeduncularis and basilar artery (1); posterior/dorsal: floor of the fourth ventricle (2); superior: a line was drawn from the floor of the fourth ventricle below the superior cerebellar peduncle (3) along the lower limit of the cerebral peduncle, to the indentation between the pons and the midbrain (4); inferior: a line was drawn from the floor of the fourth ventricle above the inferior peduncle (6) to the upper limits of the olive and pyramid of the medulla oblongata (7), i.e. to the indentation between the pons and medulla oblongata (8); on coronal view: following anatomical boundaries of pons, which are clearly visible; on axial view: following delineation in both sagittal and coronal views, the pons is now clearly delineated, and the axial view is used for verification. [5 — posterior cerebral artery].

Because the pons is not included in the 83 regions obtained via MAPER, we delineated it manually using Analyze 8.1 (Fig. 1).

We evaluated the test–retest reliability of the quantification methods (see “PET data quantification” section) in a selection of eight bilateral ROIs in total. We chose representative regions with high CB_1_ receptor concentrations; the grey matter masked cortical structures — hippocampus, anterior cingulate gyrus, and inferior frontal gyrus; and the subcortical structures in their entirety, i.e. not grey matter masked (Heckemann et al., 2011) — caudate nucleus, globus pallidus, and nucleus accumbens. In addition, two regions with low concentration of CB_1_ receptors were evaluated: the thalamus and the manually defined (entire) pons. The data from left and right homologues were averaged prior to quantification.

### PET data quantification

All dynamic PET images were de-noised and corrected for movements frame-by-frame using wavelets in Piwave 8.0 (Studholme et al., 1996, Turkheimer et al., 1999). The frame starting at 4 min (frame 10) was used as reference due to its high signal-to-noise ratio and likelihood of subjects staying still during the first minutes of the scan. The first 93 s (frames 1 to 6) were not motion corrected due to their low signal-to-noise ratio. The remaining frames (7 to 35) were automatically re-sliced and re-concatenated into a new dynamic image (Hammers et al., 2007a).

A binary contiguous mask encompassing the entire brain and extending approximately 10 mm beyond the outer cortical boundary was created semi-automatically using Analyze 8.1 and applied to both dynamic and summed radioactivity-weighted images (ADD images) to reduce computation time.

Regional quantification of distribution/binding/uptake was then performed. In all following analyses, assessment was based on the same ROIs. Binding parameters were calculated directly for the ROI TAC data, except in the “classic” spectral analysis, where the additional aim was the assessment of the quality of parametric maps for use in voxel-by-voxel analyses, and the parametric map itself was sampled using the same ROIs:

1. Compartmental models, requiring arterial IFs (section “Compartmental models, requiring 1 arterial IFs”):
  • Reversible two-compartment (one tissue compartment) model with variable blood volume (2kbv) (section “Reversible two-compartment (one tissue compartment) model with two rate constants and a variable blood volume (2kbv)”)
  • Reversible three-compartment (two tissue compartment) model with variable blood volume (4kbv) (section “Reversible three-compartment (two tissue compartment) model with four rate constants and a variable blood volume (4kbv)”)
2. Model-free analyses, requiring arterial IFs (section “Model-free analyses, requiring arterial IFs”):
  • “Classic” (non-regularised) spectral analysis (SA), applied to ROI time-activity data (section ‘“Classic” (non-regularised) SA’).
  • “Classic” SA, applied on a voxel-by-voxel basis to create parametric maps of *V*_T_, which were then sampled in the same ROIs as for the other methods (section “Sampling parametric VT images obtained voxel-by-voxel using spectral analysis (“Classic” SA)”).
  • Rank shaping regularisation of spectral analysis (SA; section “Directly obtaining VT values from ROI data with SA and rank shaping regularisation”)
3. Methods not requiring arterial IFs (section “Methods not requiring an arterial IF”):
  • Simplified reference tissue model (SRTM) using pons as a pseudo-reference tissue (section “Simplified reference tissue model using pons as a pseudo-reference tissue”)
  • (Regional) modified standard uptake values (mSUVs) (section “Modified standard uptake values (mSUVs)”)

#### Compartmental models, requiring arterial IFs

##### Reversible two-compartment (one tissue compartment) model with two rate constants and a variable blood volume (2kbv)

In this model, three microparameters are derived: K1 is the influx of the ligand from the plasma to the tissue compartment containing free, non-specifically bound, and specifically bound ligand, k2 is the efflux constant from the ROI back to plasma (Cunningham et al., 1991, Huang et al., 1980), and bv is a variable blood volume term. *V*_T_ is then calculated according to the compartmental model equation (Watabe et al., 2006):

(1) $$V_{T}=K_{1}/k_{2}.$$

##### Reversible three-compartment (two tissue compartment) model with four rate constants and a variable blood volume (4kbv)

K1 and k2 were calculated as for the 2kbv compartmental model described above; in addition, two additional rate constants were estimated to describe transfer relating to the third compartment: k3, which describes the transfer from the free and non-specifically bound compartment to the specifically bound (third) compartment; and k4, which describes the opposite transfer (Cunningham et al., 1991, Huang et al., 1980). Again, a variable blood volume term was also computed. According to compartmental model equations (Gunn et al., 2001, Watabe et al., 2006):

(2) $$V_{T}=K_{1}/k_{2}\left(1+k_{3}/k_{4}\right).$$

#### Model-free analyses, requiring arterial IFs

##### “Classic” (non-regularised) SA

Volumes of distribution (*V*_T_s) (Innis et al., 2007) for each ROI were obtained from the dynamic images and the metabolite-corrected IFs using spectral analysis (SA) (Cunningham and Jones, 1993, Cunningham et al., 1993, Turkheimer et al., 1994). The fast frequency boundary was kept at the default value of 0.1 s^− 1^. The theoretical slow frequency boundary is given by the decay constant of ^11^C (t½ ≈ 20 min, decay constant 0.0005663 s^− 1^; log_10_ = − 3.25). Based on previous work with another tracer with relatively slow kinetics (Hammers et al., 2007a), we changed this to 0.00063 s^− 1^ (log_10_ = − 3.20) in order to reduce noise.

##### Sampling parametric *V*_T_ images obtained voxel-by-voxel using spectral analysis (“Classic” SA)

Parametric images of [^11^C]MePPEP *V*_T_ were obtained from the dynamic images and the metabolite-corrected IFs using spectral analysis (SA; Cunningham and Jones, 1993, Cunningham et al., 1993) and receptor parametric mapping software (RPM6; Aston et al., 2001, Gunn et al., 1997) with the same fast and slow frequency boundaries as above. The resulting parametric maps of *V*_T_ values were then sampled in the eight selected ROIs.

##### Directly obtaining *V*_T_ values from ROI data with SA and rank shaping regularisation

*V*_T_ values were generated directly from dynamic data sampled using ROIs with rank shaping (RS; orthogonalized-functional-base) regularisation of SA (Turkheimer et al., 2003) using in-house “Clickfit” software. As previously described by Turkheimer et al. (2003), we used the metabolite-corrected IF, a logarithmically spaced basis, an exponential range of bases extending to − 3.2, and the regional tissue TAC. TACs were weighted according to (Gunn et al., 1998):

(3) $$W_{i}=L_{i}/T_{i}\left(\mathrm{for}\;\mathrm{frame}\;i=1,2,3\ldots 35\right)$$

[w_i_ — weight for frame i; L_i_ — length of frame i (seconds); and T_i_ — rate of true coincidences (per second)].

**Fig. 2:**
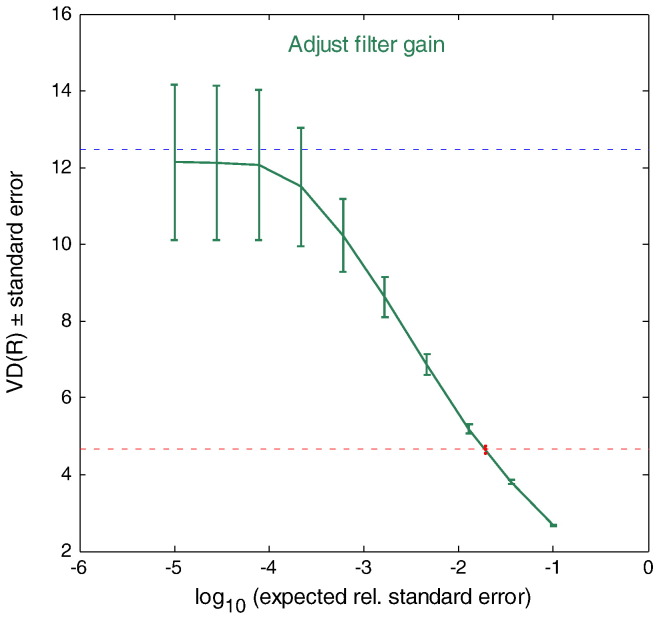
Example plot showing a *V*_T_ of 12 obtained for the hippocampus from the *V*_T_(R) plateau (see section "Directly obtaining VT values from ROI data with SA and rank shaping regularisation").

The *V*_T_ for each ROI was then obtained as the plateau of the *V*_T_(R), where R is the expected signal-to noise ratio that is used as the regularisation parameter (R) in rank-shaping (Fig. 2; (Turkheimer et al., 2003)).

#### Methods not requiring an arterial IF

##### Simplified reference tissue model using pons as a pseudo-reference tissue

CB_1_ receptors are widespread in the brain, and a true reference region devoid of specific binding does not exist. A recent attempt to obviate the invasive procedure of arterial cannulation has been to use the ROI with the lowest receptor concentration as a pseudo-reference region (Turkheimer et al., 2012). One of the structures with a low concentration of CB_1_ receptors is the pons (Yasuno et al., 2008). We therefore used the pons as a pseudo-reference region in the simplified reference tissue model (SRTM).

##### Modified standard uptake values (mSUVs)

Modified standard uptake values (mSUVs; Innis et al., 2007) for frames 25–31, i.e. from 29 min 50 s to 58 min 50 s after injection, were also derived for the ROIs according to (Goffin et al., 2011):

(4) $$\left(\mathrm{activity}\times \left[\mathrm{weight}\left(\mathrm{kg}\right)+70\;\mathrm{kg}\right]/2\right)/\mathrm{injected}\;\mathrm{dose}.$$

### Global intensities (GI)

Global intensities (GI) were calculated with an in-house script derived from SPM (Hammers et al., 2007b), where the GI is defined as the mean voxel value within a mask defined as all voxels exceeding 1/8 of the mean value of all voxels in the image matrix.

### Statistical analyses

For statistical testing we used SPSS© for Windows version 16 software (IBM 2008, New York, U.S.A.).

Injectate data were compared between test and retest sessions using the non-parametric Wilcoxon signed-rank test.

The percentage test–retest difference of parameters obtained with the various methods was calculated for all subjects in ROIs according to:

(5) $$\frac{2\cdot \left(\mathrm{retest}\;\mathrm{value}-\mathrm{test}\;\mathrm{value}\right)}{\mathrm{test}\;\mathrm{value}+\mathrm{retest}\;\mathrm{value}}\cdot 100.$$

Reliability was calculated using intraclass correlation coefficients (Maclennan, 1993):

(6) $$\mathrm{ICC}=\frac{\mathrm{MSBS}-\mathrm{MSWS}}{\mathrm{MSBS}+\left(\mathrm{df}\mathrm{WS}\times \mathrm{MSWS}\right)}$$

where MS = mean sum of squares, BS = between-subjects, WS = within-subjects, and *df* = degrees of freedom as computed in SPSS, using the “one-way random” model and reporting the “single measures” ICC. ICC values ≥ 0.75 were considered indicators of good reliability (Portney and Watkins, 2009).

We used a Bland–Altman plot to compare methods and assess bias. In the Bland–Altman plot (also known as Tukey mean-difference plot), the average of two *V*_T_ measures for the same region is plotted on the x-axis, whereas the first *V*_T_ minus the second *V*_T_ is plotted on the y axis.

$$V_{T}\left(x,y\right)=\left(\frac{V_{T1}+V_{T2}}{2},V_{T1}-V_{T2}\right)$$

Where quantification from two methods is in close agreement and without bias, the datapoints will be scattered close to y = 0, equally above and below the x axis. In this case, the 2kbv model was used as the reference, i.e. *V*_T2_ was always the average derived from 2kbv.

The association between percentage test–retest difference and interscan interval was quantified for the method yielding the highest ICCs using Spearman's rho correlation coefficient, with correction for multiple comparisons (8 regions) using the Bonferroni method. The volumes of distribution between genders were also compared using a repeated measures full-factorial general linear model, for the same method.

## Results

### Injectate

Details are given in Table 1. There were no significant differences between test and retest studies in terms of the amount of injected radioactivity (median (i.q.r)): test 362 (358–368) MBq; retest 366 (360–372) MBq; co-injected mass of stable ligand: test: 3.2 (2.4–4.1) μg, retest 3.3 (2.7–4.7) μg; and specific activity at the time of injection: test 50 (49–61) MBq/ηmol, retest: 51 (37–61) MBq/ηmol.

### Image data

**Fig. 3:**
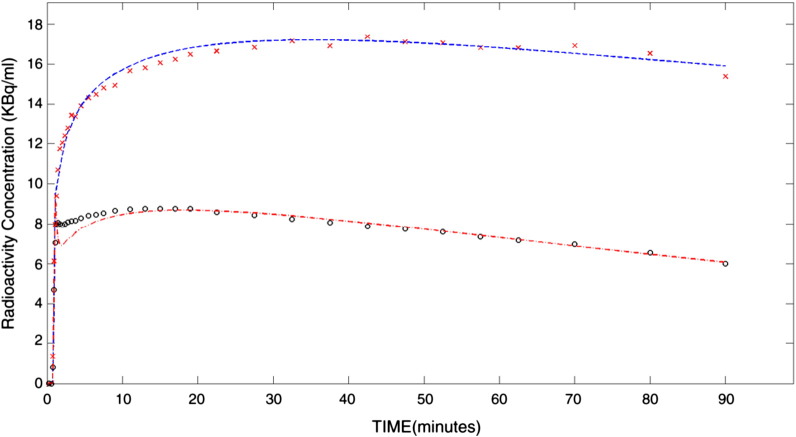
Average time activity curves for the pallidum (top) and pons (bottom): the average uptake in each region as a function of time in minutes, subsequently fitted with a two-compartment (2kbv) model.

Global intensities of summed radioactivity images did not differ between test and retest studies (median, interquartile 25th–75th: test: 1.2, 1.0–1.6; retest: 1.3, 1.1–1.5; *p > 0.6*). Fig. 3 shows examples of time–activity curves.

### Quantification results

The following sections describe the regional estimates for the parameters derived with the seven quantification methods. To assess test–retest variation, for each ROI the median percent difference between test and retest studies as well as their signed range is given in the tables. The coefficient of variation (standard deviation divided by the mean) quantifies the between-subject variability of the measure. The ICC assesses the reliability of the measure as a function of both within-subject variability and between-subjectvariability; the closer to the value of 1, the more reliable the method, i.e. the smaller the intra-subject variability of the measure compared with natural between-subject variability. Finally, the ratio of the generally highest binding region (pallidum) over the lowest binding region (pons) assesses a method's ability to reflect known binding heterogeneity.

A comparison of analysis methods and a synthetic overview of the various measures will be provided in the section “Comparison between analysis methods”.

#### Compartmental models, requiring arterial IFs

##### Reversible two-compartment (one tissue compartment) model with variable blood volume (2kbv)

The region with the highest *V*_T_ was globus pallidus (10.8; Table 2). Regional heterogeneity of *V*_T_ values was high, with a ratio of pallidum:pons of 3.6. ICCs ranged between 0.69 and 0.95, with a mean ± SD of 0.82 ± 0.09.

**Table 2:** Subjects' volume of distribution (*V*_T_) obtained with two-compartment model (2kbv) method. ACG = anterior cingulate gyrus, IFG = inferior frontal gyrus, caudate = caudate nucleus, pallidum = globus pallidus, accumbens = nucleus accumbens. BS = between-subjects, CV = coefficient of variation, diff = difference, ICC = intraclass correlation coefficient, Min = minimum, Max = maximum, ROI = region-of-interest, SD = standard deviation.

ROI	Median	Interquartile range	Min	Max	Median % diff	Median % diff range	Mean BS % CV	ICC
Hippocampus	8.0	6.2–10.8	4.1	24.8	14.8	− 54.8–54.2	50.4	0.88
ACG	10.3	7.5–14.0	1.0	26.4	27.8	− 154.4–64.2	47.4	0.76
IFG	8.8	7.3–12.3	4.1	22.4	18.1	− 48.3–31.9	42.1	0.83
Caudate	7.4	6.0–12.2	3.9	22.9	19.7	− 60.9–33.5	48.0	0.85
Pallidum	10.8	8.5–14.2	1.1	32.6	20.6	− 158.7–44.1	48.1	0.70
Accumbens	8.4	7.2–13.2	1.2	31.7	35.8	− 150.5–51.5	53.7	0.69
Thalamus	5.1	4.4–7.3	2.9	10.9	12.6	− 35.5–28.5	35.9	0.89
Pons	3.0	2.8–3.9	2.0	6.2	8.3	− 10.7–20.2	30.2	0.95
				Mean (SD)	19.7 (8.7)		44.5 (7.9)	0.82 (0.09)

##### Reversible three-compartment (two tissue compartment) model with variable blood volume (4kbv)

The method yielded highly variable data. Unlike for the other methods, the regions with the highest *V*_T_ values were nucleus accumbens (13.6; Table 3) and hippocampus (13.4). The ratio of pallidum/pons was 2.0. ICCs ranged between − 0.13 and 0.50, with a mean ± SD of 0.14 ± 0.25.

**Table 3:** Subjects' *V*_T_ obtained with three-compartment model (4kbv). ACG = anterior cingulate gyrus, IFG = inferior frontal gyrus, caudate = caudate nucleus, pallidum = globus pallidus, accumbens = nucleus accumbens. BS = between-subjects, CV = coefficient of variation, diff = difference, ICC = intraclass correlation coefficient, Min = minimum, Max = maximum, ROI = region-of-interest, SD = standard deviation.

ROI	Median	Interquartile range	Min	Max	Median % diff	Median % diff range	Mean BS % CV	ICC
Hippocampus	13.4	7.5–19.6	− 0.7	102.2	72.8	− 219.9–175.6	81.1	− 0.05
ACG	9.0	5.8–13.6	1.4	65.6	53.7	− 136.2–126	81.4	0.23
IFG	8.9	4.7–11.2	0.8	18.6	61.1	− 159–116.4	51.9	0.13
Caudate	6.2	4.2–8.5	0.0	31.5	68.5	− 153.4–200.0	69.9	− 0.13
Pallidum	10.6	6.7–18.3	3.4	79.8	69.7	− 170.7–126.1	111.3	− 0.03
Accumbens	13.6	8.7–39.5	0.0	163.5	122.1	− 72.3–200.0	131.7	− 0.06
Thalamus	6.1	4.5–8.4	1.2	30.5	32.7	− 133.8–102.5	87.9	0.49
Pons	5.3	3.9–7.2	2.4	23.0	40.3	− 23.4–146.5	79.0	0.50
				Mean (SD)	65.1 (27.1)		86.8 (24.6)	0.14 (0.25)

#### Model-free analyses, requiring arterial IFs

##### Directly obtaining *V*_T_ values from ROI data with “classic” spectral analysis (SA)

The region with the highest *V*_T_ values was globus pallidus (15.7; Table 4). Regional heterogeneity of *V*_T_ values, estimated as the ratio of pallidum over pons, was 2.6. ICCs ranged between 0.67 and 0.87, with a mean ± SD of 0.76 ± 0.07.

**Table 4:** Subjects' volume of distribution (*V*_T_) obtained with Spectral Analysis (SA) based on ROI data. ACG = anterior cingulate gyrus, IFG = inferior frontal gyrus, caudate = caudate nucleus, pallidum = globus pallidus, accumbens = nucleus accumbens. BS = between-subjects, CV = coefficient of variation, diff = difference, ICC = intraclass correlation coefficient, Min = minimum, Max = maximum, ROI = region-of-interest, SD = standard deviation.

ROI	Median	Interquartile range	Min	Max	Median % diff	Median % diff range	Mean BS % CV	ICC
Hippocampus	12.4	9.4–17.2	6.6	23.4	10.9	− 39.3–29.6	34.1	0.87
ACG	14.4	11.9–19.9	7.3	27.1	12.4	− 64.3–39.3	35.0	0.74
IFG	13.9	10.1–19.3	5.4	26.3	13.3	− 76.3–47.3	39.0	0.71
Caudate	13.0	9.3–19.4	5.9	28.1	13.6	− 80.8–26.3	44.2	0.74
Pallidum	15.7	12–21.7	8.2	28.9	9.4	− 53.8–33.5	35.5	0.85
Accumbens	13.9	11.3–18.9	6.9	26.7	15.1	− 63.6–47.1	35.2	0.80
Thalamus	9.6	6.9–12.2	4.8	20.3	24.7	− 60.7–49.2	39.4	0.70
Pons	6.0	4.6–7.1	2.9	11.8	24.8	− 62.0–39.9	35.5	0.67
				Mean (SD)	15.5 (6.0)		37.2 (3.4)	0.76 (0.07)

##### Sampling parametric *V*_T_ images obtained with “classic” spectral analysis

**Fig. 4:**
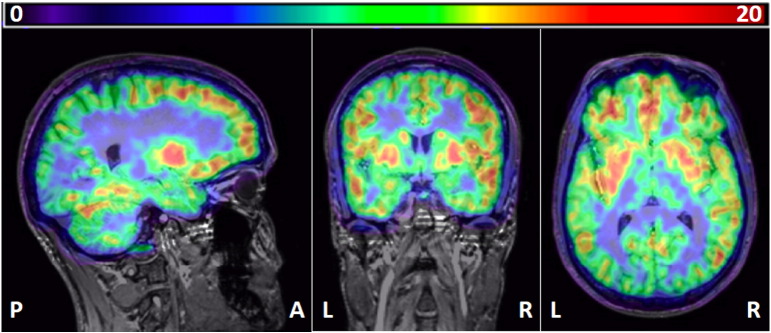
Example of a parametric map, co-registered onto the subject's MRI scan. Left, sagittal; middle, coronal; right, transverse. Note high binding in the putamen and pallidum and intermediate binding in the hippocampus and thalamus. Colour bar: *V*_T_ values. P, posterior; A, anterior; R, right; L, left.

The region with the highest *V*_T_ was the pallidum (15.8; Table 5). Regional heterogeneity of *V*_T_ values was moderate, with a ratio of pallidum over pons of 3.6. ICCs were fairly homogenous between regions and ranged between 0.76 and 0.87, with a mean ± SD of 0.83 ± 0.03. Fig. 4 shows an example of a parametric map.

**Table 5:** Subjects' volume of distribution (*V*_T_) obtained with “classic” spectral analysis (SA) on parametric maps. ACG = anterior cingulate gyrus, IFG = inferior frontal gyrus, caudate = caudate nucleus, pallidum = globus pallidus, accumbens = nucleus accumbens. BS = between-subjects, CV = coefficient of variation, diff = difference, ICC = intraclass correlation coefficient, Min = minimum, Max = maximum, ROI = region-of-interest, SD = standard deviation.

ROI	Median	Interquartile range	Min	Max	Median % diff	Median % diff range	Mean BS % CV	ICC
Hippocampus	11.5	9–16.2	7.0	22.6	9.6	− 40.1–20.6	34.2	0.87
ACG	14.8	11.1–20.5	8.1	28.8	12.5	− 44.0–30.1	34.7	0.85
IFG	14.8	10.4–20.3	7.6	27.6	12.0	− 49.7–27.0	37.0	0.85
Caudate	12.9	10.2–19.3	6.5	26.9	12.3	− 76.6–24.3	40.0	0.81
Pallidum	15.8	11.6–21.1	8.6	30.4	12.6	− 53.0–23.5	35.4	0.83
Accumbens	9.5	7.8–13.9	5.3	18.7	12.3	− 44.6–31.4	36.0	0.83
Thalamus	13.2	10.4–17.3	5.2	26.5	16.8	− 78.9–50.8	37.1	0.76
Pons	4.4	3.5–5.3	1.7	9.6	20.2	− 33.1–43.3	39.0	0.81
				Mean (SD)	13.5 (3.3)		36.7 (2.0)	0.83 (0.03)

##### Spectral analysis with rank shaping regularisation

The region with the highest *V*_T_ values was globus pallidus (10.3; Table 6). Regional heterogeneity of *V*_T_ values was lower than with the preceding methods, with a ratio of pallidum over pons of 2.4. ICCs ranged between 0.73 and 0.90, with a mean ± SD of 0.82 ± 0.05.

**Table 6:** Subjects' Volume of distribution (*V*_T_) obtained with Rank Shaping (RS) regularisation of Spectral Analysis (SA) method. ACG = anterior cingulate gyrus, IFG = inferior frontal gyrus, caudate = caudate nucleus, pallidum = globus pallidus, accumbens = nucleus accumbens. BS = between-subjects, CV = coefficient of variation, diff = difference, ICC = intraclass correlation coefficient, Min = minimum, Max = maximum, ROI = region-of-interest, SD = standard deviation.

ROI	Median	Interquartile range	Min	Max	Median % diff	Median % diff range	Mean BS % CV	ICC
Hippocampus	8.2	5.8–10.4	4.2	15.2	14.4	− 48.5–34.9	35.9	0.81
ACG	9.9	7.6–13.7	5.5	18.8	14.6	− 29.7-32.4	34.0	0.86
IFG	9.9	7.2–13.8	5.3	18.2	15.6	− 39.5-29.0	34.8	0.85
Caudate	8.1	6.1–12.6	4.4	18.1	11.6	− 88.7-27.0	42.2	0.82
Pallidum	10.3	7.0–13.0	4.5	20.7	23.3	− 64.1-42.3	39.7	0.77
Accumbens	8.6	6.1–10.5	3.2	15.9	18.5	− 77.8-58.4	36.5	0.73
Thalamus	6.5	5.5–9.4	3.5	12.7	14.9	− 43.9-31.0	34.7	0.85
Pons	4.3	3.3–5.3	2.5	8.0	7.4	− 30.6-24.1	32.3	0.90
				Mean (SD)	15.0 (4.7)		36.3 (3.2)	0.82 (0.05)

#### Methods not requiring an arterial IF

##### SRTM with pons as a pseudo-reference tissue

The method yielded inconsistent data. The regions with the highest values were the globus pallidus, the anterior cingulate gyri, and the nucleus accumbens (BP_ND_ = 1.1; Table 7). The ratio of pallidum over pons could not be calculated, as the BP_ND_ of pons as the reference region is ~ 0. ICCs ranged between − 0.29 and 0.50, with a mean ± SD of 0.07 ± 0.27.

**Table 7:** Subjects' binding potential (BP_ND_) obtained with the SRTM and pons as a pseudo-reference region. ACG = anterior cingulate gyrus, IFG = inferior frontal gyrus, caudate = caudate nucleus, pallidum = globus pallidus, accumbens = nucleus accumbens. BS = between-subjects, CV = coefficient of variation, diff = difference, ICC = intraclass correlation coefficient, Min = minimum, Max = maximum, ROI = region-of-interest, SD = standard deviation.

ROI	Median BP_ND_	Interquartile range	Min	Max	Median % diff.	Median % diff range	Mean %BS-CV	ICC
Hippocampus	1.0	0.8–1.7	0.4	12.8	54.6	− 155.5–176.6	125.2	− 0.06
ACG	1.1	0.9–1.2	0.7	3.4	40.2	− 142.9–71.7	− 574.1	0.50
IFG	0.9	0.9–1.0	0.0	1.7	16.0	− 66.6–207.6	29.4	− 0.29
Caudate	0.9	0.6–1.0	0.2	2.7	30.5	− 137.5–45.4	48.8	0.06
Pallidum	1.1	1.0–1.3	0.1	1.6	9.4	− 37.9–158.4	25.1	0.27
Accumbens	1.1	0.7–1.6	− 0.2	18.2	53.0	− 213.9–180.6	157.4	− 0.15
Thalamus	1.0	0.5–6.2	0.4	10.4	42.3	− 169.8–181.2	108.2	0.19
				Mean (SD)	35.1 (17.4)			0.07 (0.27)

##### Modified standard uptake values (mSUVs)

The region with the highest values was globus pallidus (3.6; Table 8). Regional heterogeneity of values was the highest of all methods tested with the ratio of pallidum over pons 4.2. ICCs ranged from 0.47 to 0.86, with a mean ± SD of 0.79 ± 0.13.

**Table 8:** Subjects' modified standard uptake values (mSUV). ACG = anterior cingulate gyrus, IFG = inferior frontal gyrus, caudate = caudate nucleus, pallidum = globus pallidus, accumbens = nucleus accumbens. BS = between-subjects, CV = coefficient of variation, diff = difference, ICC = intraclass correlation coefficient, Min = minimum, Max = maximum, ROI = region-of-interest, SD = standard deviation.

ROI	Median	Interquartile range	Min	Max	Median % diff.	Median % diff range	Mean %BS-CV	ICC
Hippocampus	2.7	2.1–3.2	1.8	4.2	8.3	− 22.6–34.4	27.4	0.84
ACG	3.2	2.6–3.8	2.0	5.2	8.8	− 24.1–32.7	27.2	0.86
IFG	3.2	2.6–3.7	2.1	5.3	8.9	− 22.4–35.1	27.3	0.86
Caudate	2.9	2.5–3.9	1.9	5.1	7.6	− 58.1–30.3	30.1	0.79
Pallidum	3.6	2.7–4.1	2.2	5.5	11.1	− 28.6–30.9	27.1	0.84
Accumbens	3.0	2.4–3.6	1.5	4.7	6.7	− 65.7–33.7	27.9	0.80
Thalamus	2.7	2.2–3.2	1.7	4.3	8.1	− 16.7–38.1	25.8	0.83
Pons	0.9	0.6–1.3	0.3	3.4	36.7	7.3–108.2	62.6	0.47
				Mean (SD)	12.0 (10.1)		31.9 (12.5)	0.79 (0.13)

### Comparison between analysis methods

**Fig. 5:**
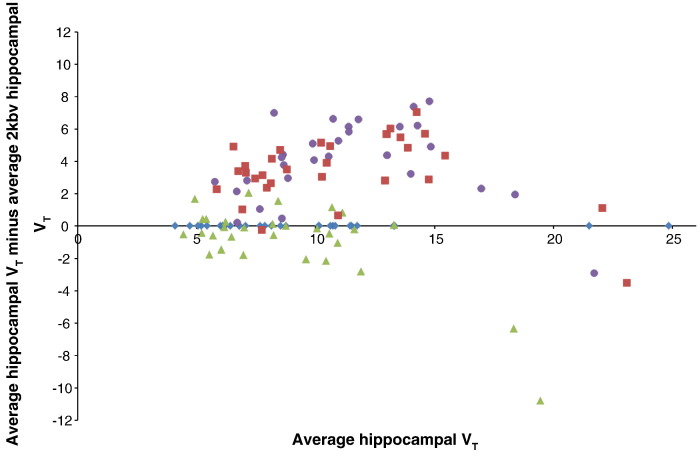
Bland–Altman plot for the different methods to obtain *V*_T_s. Data for the hippocampus is shown as an example, relative to the one compartment, two-rate constant model (blue diamonds, 2kbv); green triangles: rank-shaping regularisation of spectral analysis; red squares: “classic” voxel-wise SA; and purple circles: “classic” SA on ROI data.

Relative to the 2kbv model, a bias towards overestimation of medium high hippocampal *V*_T_ of [^11^C]MePPEP was seen for both analyses using “classic” SA (Fig. 5). RS-SA did not show this bias but restricted the range of *V*_T_ estimates, with an underestimation of the highest *V*_T_s. The 4kbv model was not assessed due to its lack of reliability.

Table 9 provides an overview of the median test–retest differences (%) for the different methods. 2kbv and the methods using SA had median differences between 13 and 20%, similar across regions as indicated by low spreads (SDs), whilst mSUVs varied even less on average, but with more between-region variation due to the pons showing high test–retest differences (37%). 4kbv and SRTM were very variable for most regions.

**Table 9:** Median test–retest differences (%) for subjects' parameter estimates (*V*_T_/BP_ND_/mSUV) obtained with the different methods. ACG = anterior cingulate gyrus, IFG = inferior frontal gyrus, caudate = caudate nucleus, pallidum = globus pallidus, accumbens = nucleus accumbens. ROI = region-of-interest, SD = standard deviation.

Method	2kbv	4kbv	SA-ROIs	SA-maps	RS-SA	SRTM	mSUV
Parameter	*V*_T_	*V*_T_	*V*_T_	*V*_T_	*V*_T_	BP_ND_	kBq/ml
Sampling	ROI on dynamic images	ROI on dynamic images	ROI on dynamic images	ROI on parametric maps	ROI on dynamic images	ROI on dynamic images	ROI on summed images
Hippocampus	14.8	72.8	10.9	9.6	14.4	54.6	8.3
ACC	27.8	53.7	12.4	12.5	14.6	40.2	8.8
IFG	18.1	61.1	13.3	12.0	15.6	16.0	8.9
Caudate	19.7	68.5	13.6	12.3	11.6	30.5	7.6
Pallidum	20.6	69.7	9.4	12.6	23.3	9.4	11.1
Accumbens	35.8	122.1	15.1	12.3	18.5	53.0	6.7
Thalamus	12.6	32.7	24.7	16.8	14.9	42.3	8.1
Pons	8.3	40.3	24.8	20.2	7.4	NA	36.7
Mean ± SD	19.7 ± 8.7	65.1 ± 27.1	15.5 ± 5.9	13.5 ± 3.3	15.0 ± 4.6	35.1 ± 17.4	12.0 ± 10.1

Mean between-subject coefficients of variation (BSCV; %) for the different methods are listed in Table 10. The between-subject variability based on mSUVs (i.e. tissue data only) was approximately 32%; however the BSCV for pons was 63%. The three methods based on SA had similar between-subject variation, around 36%, with similar variability for the various regions. Between-subject variability was higher for the 2kbv compartmental model, at 45%. SRTM and the 4kbv model yielded implausible values.

**Table 10:** Mean between-subject coefficients of variation (BSCV; %) for subjects' *V*_T_/BP_ND_/mSUV parameters obtained with the different methods. ACG = anterior cingulate gyrus, IFG = inferior frontal gyrus, caudate = caudate nucleus, pallidum = globus pallidus, accumbens = nucleus accumbens. ROI = region-of-interest, SD = standard deviation, NA = not applicable.

Method	2kbv	4kbv	SA-ROIs	SA-maps	RS-SA	SRTM	mSUV
Parameter	*V*_T_	*V*_T_	*V*_T_	*V*_T_	*V*_T_	BP_ND_	kBq/ml
Sampling	ROI on dynamic images	ROI on dynamic images	ROI on dynamic images	ROI on parametric maps	ROI on dynamic images	ROI on dynamic images	ROI on summed images
Hippocampus	50.4	81.1	34.1	34.2	35.9	125.2	27.4
ACG	47.4	81.4	35.0	34.7	34.0	− 574.1	27.2
IFG	42.1	51.9	39.0	37.0	34.8	29.4	27.3
Caudate	48.0	69.9	44.2	40.0	42.2	48.8	30.1
Globus Pallidum	48.1	111.3	35.5	35.4	39.7	25.1	27.1
Accumbens	53.7	131.7	35.2	36.0	36.5	157.4	27.9
Thalamus	35.9	87.9	39.4	37.1	34.7	108.2	25.8
Pons	30.2	79.0	35.5	39.0	32.3	NA	62.6
Mean ± SD	44.5 ± 7.9	86.8 ± 24.6	37.2 ± 3.4	36.7 ± 2.0	36.3 ± 3.2	− 11.4 ± 253.2	31.9 ± 12.5

ICCs for all methods are listed in Table 11. As expected from the high test–retest variability (Table 9) and unrealistically high between-subject variability (Table 10), the 4kbv model and SRTM yielded non-reproducible results, as reflected by an ICC around zero. All the other methods had good to very good reproducibility, ranging from 0.76 ± 0.07 for classic SA calculated on ROI data to 0.83 ± 0.04 for classic SA applied to parametric maps. Note the low between-region spread of the ICC for the five methods with good or very good reproducibility, meaning that reproducibility was good throughout the brain regions sampled.

**Table 11:** Intraclass correlation coefficients (ICCs) for subjects' volume of distribution (*V*_T_) and/or binding potential (BP) obtained with the different methods. The ratio *V*_T_/BP of a high binding region (pallidum) over that of a low-binding region (pons) is also given. ACG = anterior cingulate gyrus, IFG = inferior frontal gyrus, caudate = caudate nucleus, pallidum = globus pallidus, accumbens = nucleus accumbens. ROI = region-of-interest, SD = standard deviation, NA = not applicable.

Method	2kbv	4kbv	SA-ROIs	SA-maps	RS-SA	SRTM	mSUV
Parameter	*V*_T_	*V*_T_	*V*_T_	*V*_T_	*V*_T_	BP_ND_	kBq/ml
Sampling	ROI on dynamic images	ROI on dynamic images	ROI on dynamic images	ROI on parametric maps	ROI on dynamic images	ROI on dynamic images	ROI on summed images
Hippocampus	0.88	− 0.05	0.87	0.87	0.81	− 0.06	0.84
ACG	0.76	0.23	0.74	0.85	0.86	0.50	0.86
IFG	0.83	0.13	0.71	0.85	0.85	− 0.29	0.86
Caudate	0.85	− 0.13	0.74	0.81	0.82	0.06	0.79
Pallidum	0.70	− 0.03	0.85	0.83	0.77	0.27	0.84
Accumbens	0.69	− 0.06	0.80	0.83	0.73	− 0.15	0.80
Thalamus	0.89	0.49	0.70	0.76	0.85	0.19	0.83
Pons	0.95	0.50	0.67	0.81	0.90	NA	0.47
Mean ± SD	0.82 ± 0.09	0.14 ± 0.25	0.76 ± 0.07	0.83 ± 0.03	0.82 ± 0.05	0.07 ± 0.27	0.79 ± 0.13
Ratio pallidum/pons	3.6	2.0	2.6	3.6	2.4	NA	4.2

Table 11 also shows the ratio between a high-binding region (pallidum) and a low-binding region (pons), indicating a method's ability to reflect the known between-region heterogeneity. mSUVs had the highest differential, followed by 2kbv model and SA applied to parametric maps.

A positive correlation was observed for test–retest difference and interscan interval in the inferior frontal gyrus (p = 0.039, Spearman's rho = 0.538) and the caudate (p = 0.033, Spearman's rho = 0.550). These correlations were rendered insignificant by correction for multiple comparisons. There was no significant influence of gender on the V_T_s (F = 27.349, p = 0.995). There was no significant interaction between gender and test–retest condition (p = 0.120).

## Discussion

We describe the test–retest reproducibility of quantification for CB_1_-receptor availability, as assessed by [^11^C]MePPEP PET, in 15 healthy human subjects. Our major finding is that good-to-excellent reproducibility of estimates of availability is achievable using either the one tissue compartment, two rate-constant kinetic model with a variable blood volume term; model-free analyses using spectral analysis variants; or simple scaled measures of radioactivity (mSUV).

The performance of the various methods was consistent between measures — those having low percentage test–retest variability also had high ICCs, reflecting that among the well-performing methods, between-subject variability was comparable.

The 2kbv compartmental model was among the best performing methods for test–retest variability and reliability, and also had one of the highest ratios of pallidum over pons. This indicates low bias (i.e. a large range of concentrations between regions of known high and low receptor availability). This ratio was lower for the spectral analysis variants applied to ROI data, reflecting their known bias towards lower *V*_T_ estimates in high binding regions (Hammers et al., 2007a). In contrast, voxel-wise SA had the same high pallidum/pons ratio as the 2kbv model. This may be due to SA's ability to fit voxel-wise time courses — voxels with varying partial volume contributions of white matter or vasculature can be individually fitted, which is not the case for methods using the averaged ROI TAC.

Several, but not all (e.g. Ahmad et al., 2014, Goffin et al., 2011, Van Laere et al., 2010), earlier in vivo human studies with [^18^F]MK-9470 (Burns et al., 2007), [^11^C]MePPEP (Terry et al., 2009) and [^18^F]FMPEP-*d*_2_ (Terry et al., 2010b) involved 120 to 300 min scan times (Terry et al., 2009, Terry et al., 2010b). This requirement limits the usefulness of these authors' approaches, as patients with debilitating conditions and even healthy volunteers are unlikely to tolerate PET scans of 2 h duration or more. Here we present data that indicates that with [^11^C]MePPEP, reliable quantification of CB_1_ receptor availability is achievable with just 90 min of data acquisition. In a previous study, it has been shown that 90 min of acquisition is sufficient for obtaining stable *V*_T_ estimates (Terry et al., 2009).

In addition, the injected doses used in previous studies were generally approximately twice as high as the doses used in our study, up to 750 MBq of [^11^C]MePPEP (Terry et al., 2009). We achieved reliable receptor availability estimation using only 370 MBq, entailing an effective dose of just ~ 1.7 mSv per scan (Terry et al., 2010a). We had previously observed that both image quality and the reliability of blood data measurements were lower when injected doses of a radioligand with similarly slow kinetics were lowered to ~ 180 MBq (Hammers et al., 2007a). In our hands, mSUVs – using only tissue data – yielded excellent test–retest properties and differentiation between regions, with the highest pallidum/pons ratio of all methods. The fact that excellent reliability and differentiation between regions with high and low receptor concentrations (Herkenham et al., 1990) could be achieved with methods using metabolite-corrected arterial plasma input functions suggests the reliability of the blood measurements.

Because arterial cannulations require skilled personnel and involve discomfort and small risks to volunteers and patients, non-invasive PET studies are usually preferred in research studies, and even more so in a clinical environment. Methods using a reference region devoid of the studied receptor are needed for full quantification in the absence of an input function. However, CB_1_ receptors are present throughout the brain, and a true reference region does not exist. Here we used the pons as a pseudo-reference region. It has low CB_1_ receptor concentration (Herkenham et al., 1990, Irving et al., 2002, Terry et al., 2009, Yasuno et al., 2008), motivating this attempt despite some specific binding (Terry et al., 2009, Yasuno et al., 2008). We were unable to achieve reliable data. The application of more sophisticated pseudo-reference region approaches as described in recent studies (Turkheimer et al., 2012) might improve on these results. However, we note that the pons tissue data (i.e. mSUV) measurements were far less reliable than measurements elsewhere. Even small variations in the amount of specific binding between individuals may thus have a large influence on the radioactivity concentration in this region, with resulting low reliability for the SRTM.

This is the first study to apply model-free analyses (spectral analysis with or without rank shaping regularisation) to quantify cannabinoid receptor availability using [^11^C]MePPEP PET. These have the advantage of being ‘data-driven’ rather than requiring an a priori model selection. We additionally describe the first voxel-wise quantification of [^11^C]MePPEP, yielding parametric *V*_T_ images with high corresponding regional ICCs. Spectral analysis does not require a priori assumptions regarding the number of components, compartments, or distribution of receptors. Spectral analysis estimations require compartmental systems that are strongly connected, have exchange of material with the environment confined to a single compartment, and do not contain cycles, i.e., there is no possibility for material to pass from one compartment through two or more compartments back to the initial compartment (Schmidt, 1999). SA cannot be used to estimate reference region models because the fit assumes a sum of positive series of convolution integrals of the input function. This last condition is relaxed in Rank-Shaping SA.

We do not expect any biases when spectral analysis is applied to data from participants who are not healthy controls. SA has been used successfully in several patient populations.

The improved ICC measures of SA methodologies over compartmental ones indicate a complex compartmentalisation of the underlying receptor distribution. CB1 receptors can be found on neurons, astrocytes, and also the vascular endothelium. Compartmental models usually assume that a single concentration represents the free-in-tissue tracer compartment. This concentration in reality is the average of concentrations of the free radioligand in different tissue environments, as there will be a gradient across different cellular elements. This will cause an apparent change in affinity of the tracer depending on the target cell (Delforge et al., 1996). Spectral analysis, which does not depend on a free compartmental structure, has more flexibility in dealing with such a complex signal.

A major difference relative to the previous test–retest study (Terry et al., 2009) is the lack of reliability of *V*_T_ estimates obtained with the two-tissue compartment model (4kbv) in our study, as well as good reliability for *V*_T_ estimates obtained using the one-tissue compartment model 2kbv, whereas this had yielded poor fits for Terry et al. (2009). This might relate to longer scanning time and nearly twice the injected dose in the former study. An additional major difference in the models is that we estimated the blood volume contribution, whereas this had been set to 5% in the previous study. Of note, for a similar time interval our standardized uptake values are comparable to those of Terry et al. (2009).

We did not collect data from the female participants concerning the stage of their menstrual cycle on the day of scanning. Here, when this was tested for the “classic” spectral analysis (parametric maps) method, there was no significant between-subject influence of gender on the *V*_T_s; nor was there a significant interaction between gender and test–retest condition. Whilst further investigation is required, we therefore hypothesise that the menstrual cycle has a minimal effect on the variability of [^11^C]MePPEP *V*_T_ in human females.

We did not calculate the parent tracer free fraction, which may be important (Terry et al., 2010a, Terry et al., 2010b, Yasuno et al., 2008). However, our result of good reproducibility with tissue-only data (mSUV) and even better reproducibility with modelling approaches using the arterial input data suggests that this omission did not have an adverse effect.

In conclusion, quantification of CB_1_ receptor availability showed good-to-excellent reproducibility with selected kinetic and model-free analyses, whether applied on a region-of-interest or voxel-wise basis. [^11^C]MePPEP PET is well-placed as a tool to investigate CB_1_ receptor-mediated neurotransmission in health and neuropsychiatric disease.
